# Association of Irisin Plasma Levels with Anthropometric Parameters in Children with Underweight, Normal Weight, Overweight, and Obesity

**DOI:** 10.1155/2017/2628968

**Published:** 2017-05-03

**Authors:** Leticia Elizondo-Montemayor, Christian Silva-Platas, Alejandro Torres-Quintanilla, Carlos Rodríguez-López, Guillermo U. Ruiz-Esparza, Eric Reyes-Mendoza, Gerardo Garcia-Rivas

**Affiliations:** ^1^Tecnológico de Monterrey, Centro de Investigación en Nutrición Clínica y Obesidad, Escuela de Medicina, 64849 Monterrey, NL, Mexico; ^2^Tecnológico de Monterrey, Centro de Investigación Biomédica, Hospital Zambrano Hellion, 66278 San Pedro Garza-García, NL, Mexico; ^3^Tecnológico de Monterrey, Cátedra de Cardiología y Medicina Vascular, Escuela de Medicina, 66278 San Pedro Garza-García, NL, Mexico

## Abstract

The correlations between irisin levels, physical activity, and anthropometric measurements have been extensively described in adults with considerable controversy, but little evidence about these relationships has been found in children. The objective of this study is to correlate the plasma levels of irisin in underweight, normal weight, overweight, and obese children with anthropometric parameters and physical activity levels. A cross-sample of 40 children was divided into the following groups on the basis of body mass index (BMI) percentile. The correlations of plasma irisin levels with physical activity, anthropometric, and metabolic measurements were determined. Plasma irisin levels (ng/mL) were lower for the underweight group (164.2 ± 5.95) than for the normal weight and obese groups (182.8 ± 5.58; *p* < 0.05). Irisin levels correlated positively with BMI percentile (0.387), waist circumference (0.373), and fat-free mass (0.353; *p* < 0.05), but not with body muscle mass (−0.027). After a multiple linear regression analysis, only BMI percentile (0.564; *p* < 0.008) showed a positive correlation with irisin. Our results indicated no association with metabolic parameters. A negative correlation with physical activity was observed. Interrelationships among body components might influence irisin levels in children.

## 1. Introduction

Obesity has become a public health issue in many countries; Mexico and the United States present the highest obesity rates in the world [1]. This issue is particularly important in obese children and adolescents, as they show increased morbidity and premature mortality because of their higher risk for chronic cardiometabolic diseases and other comorbidities [2].

Unraveling the factors that contribute to obesity is complex; however, recent research has revealed that cytokines and other peptides participate in the multifactorial pathogenic mechanisms of obesity [3]. One recently discovered myokine, irisin, has attracted special attention as a possible factor that contributes to obesity. Irisin is a hormone secreted as a cleavage product from fibronectin type III domain-containing protein 5 (FNDC5), which is normally present in the skeletal muscle of mice and humans [4, 5]. The effects of this hormone are mediated by the upregulation of uncoupling protein 1 (UCP1) during physical activity, which increases thermogenesis, energy expenditure, and browning of white adipose tissue [4].

On a clinical level, the significant correlations between irisin levels and anthropometric measurements have been described [6]. Nevertheless, considerable contradictory data that correlate plasma irisin levels in obese and anorexic individuals with different anthropometric parameters have been reported. For instance, negative associations between serum irisin levels and anthropometric and metabolic obesity markers have been found primarily in adults with the metabolic syndrome and with fasting hyperglycemia [7]. By contrast, positive associations between serum irisin and obesity markers, such as body weight, body mass index (BMI), fat mass, and fat-free mass, have been found [8–10]. Interestingly, after a dietary intervention, subjects who regained weight showed increased plasma irisin levels that were positively associated with insulin resistance [11]. Nonetheless, other groups have found evidence of null associations. For example, a study in obese individuals showed that although their irisin level increased by 12% after a year of lifestyle intervention, these were not correlated with BMI changes [12].

Contradictory findings have also been found regarding the production of irisin in human tissues. Muscular mass has been reported as the main predictor for circulating irisin, whereas in other studies, fat mass has been found as the major factor that influences irisin levels [6, 10]. These differences can be attributed to the fact that irisin secretion by subcutaneous adipocytes is dependent on the nutritional status and physical activity of the organism [13]. Lastly, researchers have found that exercise upregulates irisin secretion in mice and humans [4], a finding that was later confirmed by the same group with the use of tandem mass spectrometry [5].

While many studies have attempted to describe the role of irisin in the health of adults, the significance of irisin during childhood has often been underexplored. Few studies have focused on the relationship between irisin and markers of adiposity and physical activity in children [14]. To our knowledge, the present study is the first to examine the association of irisin with anthropometric and physical activity parameters in children with a broad spectrum of BMI percentile statuses.

## 2. Material and Methods

### 2.1. Study Population

A population comprising children aged between 6 and 12 years was selected from eight public schools in Monterrey, a city in Northeast Mexico. From the total population, 1,300 children were randomly selected with an error of <1.4%. The inclusion criteria were attendance from first to sixth grade, Mexican nationality, and a 12-hour overnight fasting. The exclusion criterion was disapproval by the children's physician because of any medical conditions that would preclude their participation in the study. Based on the sample size calculation, a cross-sample of 40 children (20 girls and 20 boys) was selected; this sample was divided into four groups according to the BMI percentile (*n* = 10/group) and screened for irisin levels. With the use of criteria defined by the World Health Organization, groups were defined as underweight for a BMI ≤ 5th percentile, normal weight for a BMI > 5th and <85th percentiles, overweight for a BMI ≥ 85th and <95th percentiles, and obese for ≥95th percentile according to age and sex [15]. The children did not follow any specific diet, just their regular diet. Approvals were obtained from the Ethics and Research Committees of the School of Medicine of Tecnológico de Monterrey and from the government state education authorities of Nuevo Leon, Mexico. The participants did not receive any form of compensation for participating in this study. All parents or legal guardians gave their written informed consent.

### 2.2. Anthropometric Measurements

Anthropometric measurements were performed on all participants at each school. Height was determined to the nearest 0.5 cm (portable Seca® stadiometer, North America), whereas weight was identified to the nearest 0.1 kg while the children wore light clothing and no socks or shoes (TANITA TBF 300® scale, Arlington, IL). Body fat% was measured by bioimpedance (same TANITA scale). Waist circumference (WC) was measured to the nearest 0.1 cm at the level of the umbilicus with the use of a flexible fiberglass tape while the subjects were standing, after gently exhaling, and with no clothing on the area. Tricipital skinfold (TSF) was measured with a Lange skinfold caliper [16], whereas mid-upper arm circumference (MUAC) (cm) was measured with a flexible fiberglass tape around the mid-upper arm at the midpoint between the acromion and the olecranon. All measurements were performed by three trained registered dietitians (RDs) to control interobserver variability. BMI was obtained by weight-kilograms, divided by the square of height-meters. MUAMC (cm) and mid-upper arm muscle area (MUAMA) (cm^2^) were determined to calculate body muscle mass. MUAMC was calculated as MUAC − [(3.14159)*∗*(TSF mm/10)] and MUAMA (cm^2^) as [(MUAMC  cm^2^)/(4*∗*3.14159)] − 10. Body muscle mass was calculated as (height-cm)[0.264 + (0.0029*∗*MUAMA-cm^2^)] [17]. Fat mass (kg) was calculated as [(weight-kg)*∗*(body  fat%)]/100. Fat-free mass (kg) was calculated as [weight-kg − (weight-kg × body fat%)] [18].

### 2.3. Physical Activity Measurement

Information on physical activity was taken from a previously validated questionnaire administered to each child and parent/caregiver at the initial face-to-face interview with the RDs; this process provided information on the children's hours per day and days per week of regular exercise [16]. As the purpose was to obtain information on regular physical activity, the children and/or their guardians were asked, during the past six months, (1) whether the children exercised; (2) if they did, what the type of exercise they engaged in was, for example, aerobic (football, basketball, volleyball, swimming, dancing, running, walking, or cycling) or anaerobic (sprinting, mountain climbing, rope-jumping, hill climbing, isometrics, or any rapid burst of hard exercise); (3) what the number of days that they engaged in physical activity per week was; and (4) what the number of hours that they exercised per day was. None of the children practiced anaerobic physical activity. All of them reported the aerobic exercise type.

### 2.4. Blood Sample Extraction and Preparation

Blood samples were obtained via venipuncture from the median cubital vein of the arm after a 12-hour overnight fasting. The samples were kept at 2°C to 8°C for further centrifugation within the first three hours. Afterward, the samples were stored at −80°C.

### 2.5. Irisin Plasma Levels and Metabolic Assessment

Plasma irisin concentration was measured with enzyme-linked immunosorbent assay (ELISA) kit (Phoenix Pharmaceuticals, Burlingame, CA) [19]. The assay has been proven to be highly sensitive to human irisin. The sensitivity of the assay was 0.2 ng/mL, and the linear range of the standard was 1 to 1000 ng/mL. The intra- and interassay variations were both less than 5%.

Albumin, glucose, triglycerides, and cholesterol (total, HDL, and LDL) were determined in serum with the use of clinical-grade reagents from Pointe Scientific (Canton, MI), following the manufacturer's instructions.

### 2.6. Statistical Analysis

The sample size was determined in consideration of the BMI percentile variation during the comparison of the four groups with the use of ANOVA with an alpha level of 0.05 and a power of 0.8; a relevant difference in irisin levels of at least 15 ng/mL and an SD of 20 ng/mL were expected. The demographic and anthropometric data in Table 1 are presented as median and range. Metabolic parameters are presented as mean ± SD. All pairwise multiple comparisons for each parameter between the underweight, normal weight, overweight, and obese groups were performed according to the previous normality test (Kolmogorov). The parametric ANOVA test (Holm-Sidak method) was applied to normal data, and the Kruskal-Wallis ANOVA on ranks (Tukey test) was applied to nonnormal data. The alpha value was set at 0.05. The correlation coefficients and significance (*p* < 0.05) in Table 2 were calculated with Pearson product moment. Multiple linear regression analysis was performed after the removal of variables showing collinearity [19]. The analyses were performed with SigmaStat 3.11 (Systat Software Inc.) and Minitab (Minitab Inc.) and the covariance analyses with the* stats* library of the R programming language (R Core Team).

## 3. Results

### 3.1. Demographic, Anthropometric, and Metabolic Parameters according to BMI Percentiles

Table 1 shows the demographic and anthropometric characteristics of each group. Each group had an equal distribution of male and female participants across the age ranges described. Body weight (kg) was significantly higher for the obese group (42.7  [33.3–63.4]) than the overweight (34.6  [24.9–49.6]), normal weight (29.3  [20.5–41.3]), and underweight groups (21.6  [17.1–29.5]). The BMI percentile was significantly lower for the underweight group (3  [1–3]) and highest for the obese group (98  [98-99]). WC (cm) and MUAC (cm) increased significantly according to the BMI percentiles of the groups. The TSF (mm) was significantly higher for the obese group (33  [25–51]) than for the normal weight (11.5  [7–16]) and underweight groups (7  [4–11]). Triglycerides (86.61 ± 9.08 mg/dL), total cholesterol (141.35 ± 11.81 mg/dL), and LDL-c (109.12 ± 11.14 mg/dL) were significantly higher for the obese group than for the other groups, whereas HDL-c, glucose, and albumin were similar among all the BMI percentile groups. None of the children in the underweight group had an albumin level lower than 3.5 g/dL.

### 3.2. Body Composition across Groups

**Figure 1:**
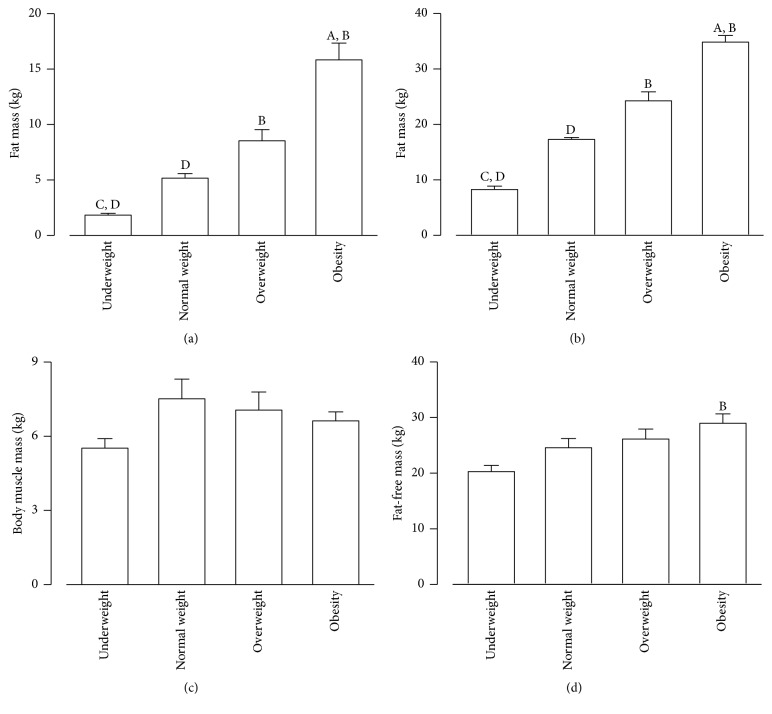
*Fat and *body muscle mass* composition according to weight status*. Group body composition parameters for (a) fat mass (kg), (b) fat mass (%), (c) body muscle mass (kg), and (d) fat-free mass (kg). Data presented as mean ± SEM; *p* < 0.05; ^A^versus normal weight, ^B^versus underweight, ^C^versus overweight, and ^D^versus obese.

Figure 1 presents the diverse elements of body composition among the groups. Fat mass (kg) and fat percentage (%) increased accordingly with the BMI and were significantly higher in the obese group than in the underweight and normal weight groups (*p* < 0.05) (Figures 1(a) and 1(b)). Body muscle mass (kg) was lowest for the underweight group (5.51 ± 0.38) and highest for the normal weight group (7.51 ± 0.79), with intermediate values for the overweight (7.05  ±  0.74) and obese groups (6.62  ±  0.36); however, these differences were not significant (Figure 1(c)). Fat-free mass (kg) was highest in the obese group (28.97 ± 1.68) and decreased accordingly in the overweight (26.12 ± 1.79), normal weight (24.55 ± 1.68), and the underweight groups (20.27 ± 1.11), although a significant difference was found only between the obese and the underweight groups (*p* < 0.05) (Figure 1(d)).

### 3.3. Irisin Plasma Levels in Groups with Different BMIs

**Figure 2:**
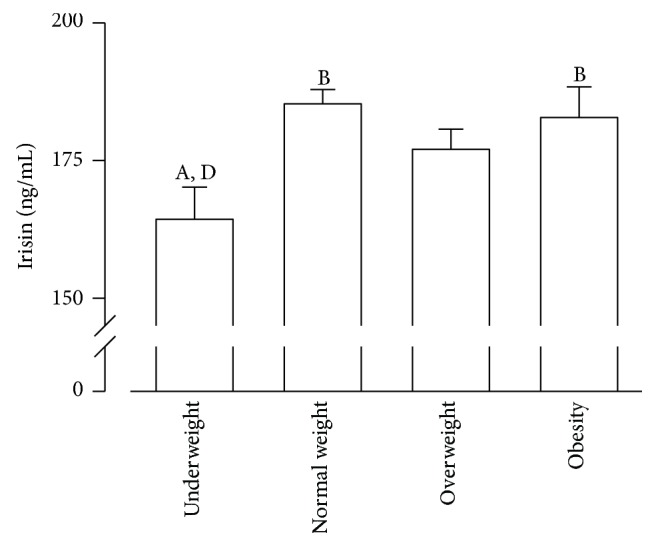
*Plasma irisin levels are reduced in underweight children*. Serum irisin levels for weight groups. Data presented as mean ± SEM; *p* < 0.05; ^A^versus normal weight, ^B^versus underweight, ^C^versus overweight, and ^D^versus obese.

The irisin plasma levels for all groups are shown in Figure 2. The irisin plasma levels (ng/mL) were significantly lower for the underweight group (164.3  ±  5.95) than for the normal weight (185.29  ±  2.62) and obese groups (182.8  ±  5.58) (*p* < 0.05). The overweight and obese groups showed no difference compared with the normal weight group.

### 3.4. Association between Irisin Plasma Levels and Body Composition

**Figure 3:**
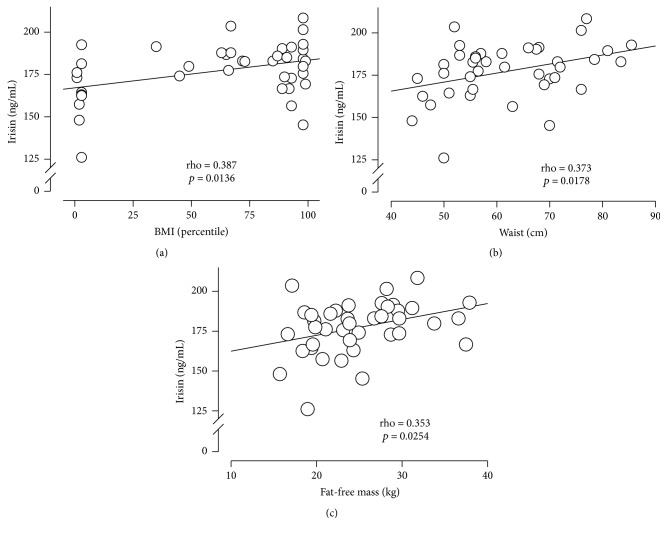
*Association of irisin plasma levels with BMI, WC, and fat-free mass*. Irisin levels according to (a) BMI percentile, (b) waist circumference (WC), and (c) fat-free mass (Kg).

The correlations of irisin plasma levels with body composition parameters are presented in Table 2. A significant Pearson correlation was found between irisin levels and BMI percentile (0.387, *p* = 0.01), WC (0.373 *p* = 0.01), and fat-free mass (0.353, *p* = 0.02). Moreover, the metabolic parameters did not show a significant correlation with the irisin levels (Supplemental Table  1 in Supplementary Material available online at https://doi.org/10.1155/2017/2628968). As previously described, sex had a significant effect on irisin levels (*p* < 0.005) [20]. Thus, after adjusting for gender, the top most significant covariate was BMI percentile (Supplemental Table 2). Together with gender, no further covariates were significant. Multiple linear regression analysis indicated that only BMI percentile (0.564; *p* < 0.008) had a positive correlation with irisin (Table 2). Furthermore, the model explains 40% of the variation in the irisin levels.  Figure 3 shows the distribution of the irisin levels per BMI percentile (Panel (a)), WC (Panel (b)), and fat-free mass (Panel (c)). Irisin levels (ng/mL) increased as the BMI percentile increased, from 125.9 to 167.8 for the 0 to 25th BMI percentile, from 167.8 to 180.4 for the 25th to 50th percentile, from 180.4 to 187.7 for the 50th to 75th percentile, and from 187.7 to 208.2 for the 75th to 100th percentile.

### 3.5. Irisin Plasma Levels and Physical Activity

**Figure 4:**
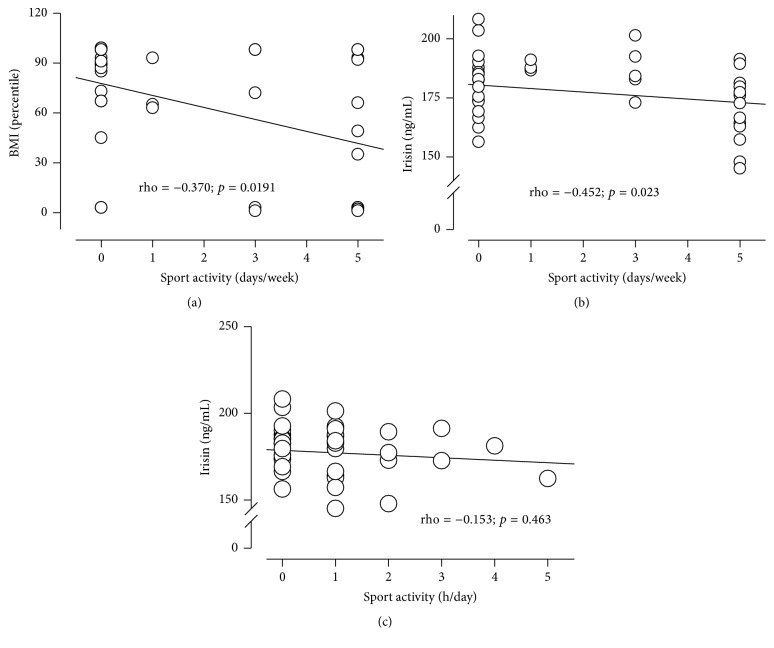
*Irisin shows negative correlation with physical activity*. (a) BMI according to physical activity. Correlation between irisin levels and physical activity in (b) (days/week) and (c) (h/day).

Figure 4 shows the association of physical activity with BMI percentile and irisin plasma levels. None of the children performed anaerobic exercise, so only aerobic exercise is reported specifically in the number of days per week and hours per day of physical activity. Underweight children exercised twice as much (3.6 ± 0.73 days per week) as overweight (1.1 ± 1.04 days per week) and obese children did (1.6 ± 0.82 days per week) (*p* < 0.01) (Figure 4(a)). Irisin showed a small but significant negative correlation with physical activity (days per week) (*p* < 0.02) (Figure 4(b)). Irisin showed a negative, although not significant, correlation with hours per day of physical activity (Figure 4(c)).

## 4. Discussion

Our measurements of adipose tissue in children showed positive correlations among them as the BMI increased, as has also been described in previous studies that related these adiposity parameters to plasma irisin levels in adult populations [8, 10, 11]. However, some may still argue that body weight, BMI, and BMI percentile do not strictly represent adiposity, as they might also include other body components and not only fat mass [21].

The body composition for each BMI percentile group in children was different. The underweight group showed the highest proportion of body muscle mass to fat mass, followed by the normal weight, overweight, and obese groups, in this order. The different body composition of each group might account for the variations in the irisin plasma levels, but this relationship remains to be defined for children [14].

Our results show that underweight children had the lowest plasma irisin levels, a finding that agrees with those of other studies that have reported higher irisin levels in obese individuals [6, 8, 10, 11, 13]. Nonetheless, some studies have described lower irisin with increased weight or BMI [7, 22, 23]. Notably, Stengel et al. reported lower plasma irisin in anorexic adult patients and a linear relationship between the irisin levels and BMI in adults [8], similar to our finding in children with underweight status. Moreover, a recent study by Palacios-González et al. [14] showed higher levels of circulating irisin in obese children and a positive correlation with BMI, also in agreement with our results.

Our results show a significant positive association of plasma irisin levels with WC, as well as a positive nonsignificant correlation with fat mass (kg) and fat mass (%). Accordingly, other studies have reported irisin associations with adiposity measurements, such as fat mass in adults [6, 8, 24]. Likewise, Pardo et al. found a twofold increase in irisin levels per 1 kg increase in fat mass [10], whereas others found no significant relationship between irisin concentrations and body muscle mass [7, 25]. Our results disagree with only one report we found in children, which involved 65 obese patients in a year-long lifestyle intervention program involving regular exercise. This report showed no correlation between irisin and markers of obesity [12].

Irisin secretion might be explained by not only the total amount of fat mass or body muscle mass but also by the relative proportion of body muscle mass to fat mass. Accordingly, we found the lowest irisin levels in the underweight group, which was also the group with the lowest total fat mass and % body fat and the highest body muscle mass to fat mass proportion. A possible explanation for the low irisin level in the underweight group is that irisin promotes the “browning” of subcutaneous white adipose tissue, capable of burning energy through uncoupling protein 1-mediated thermogenesis, and increased energy expenditure. Uncoupling protein 1 is an enzyme that uncouples oxidative phosphorylation from ATP production, leading to energy release as heat. The underweight group in this study had a lower fat mass and % fat mass. Thus, we speculate that the lower irisin levels in the underweight children may represent an adaptive response to conserved energy. In accordance with this result, Singhal et al. identified lower irisin levels in young amenorrheic athletes than in eumenorrheic athletes and nonathletes. These amenorrheic athletes presented a lower total fat mass, as well as a lower % body fat. The authors hypothesized that the irisin levels were lower as an adaptive response to an overall state of energy deficit [26].

In line with our results, several studies have shown that irisin is also released by adipocytes and is produced in a positive feedback loop [13, 22], whereas Roca-Rivada et al. found that adipose tissue is primarily responsible for irisin secretion [13]. The amount of brown adipose tissue has been found to be significantly decreased along with obesity, BMI, and % body fat [27, 28]. On the other hand, irisin has been found to enhance white adipocyte transdifferentiation into beige adipocytes by increasing UCP1 and PRDM16 and to burn lipids as fuel by increasing PGC1a and MTCO3 [22]. Therefore, we speculate that the higher irisin levels in the obese children than in the underweight ones in this study might suggest that irisin played a compensatory role in trying to increase brown adipose tissue in the obese children.

Furthermore, the distribution of fat might play a role in metabolic and cardiovascular disorders, such as insulin resistance. WC has been considered a good surrogate measurement of visceral fat [29, 30]. In previous studies in adults, WC was negatively associated with plasma irisin [7, 31]. However, we found a positive correlation between WC and irisin. Because WC has been known to be associated with insulin resistance, irisin may act as a counterregulator of insulin resistance in children, as has been suggested for adults [29, 30, 32–34].

Obese children have been found to have an increased risk for insulin resistance, the metabolic syndrome, and cardiovascular disease [3–5]. In addition, higher irisin levels in obese adults have been associated with risk factors for insulin resistance [8, 11, 24]. The latter may suggest that the obese children in our study showing higher irisin levels might have an increased risk for insulin resistance.

Furthermore, a recent study by Viitasalo et al. has shown the association between increased irisin levels and a deteriorated lipid profile in children [35]. In a recent paper on obese children, the multivariate regression analysis showed that HDL-c had a significant association with the irisin plasma level [36], but our results indicated no association of irisin with HDL-c, LDL-c, triglycerides, and total cholesterol. Our glucose values and especially HDL-c values are too low compared with those of other studies. Low HDL-c levels, similar to ours, have been found as minimal values in underweight, normal weight, overweight and obese Mexican children: low or normal weight children had a HDL-c minimum value of 14 mg/dL (media 52 + 15.2) and overweight/obese children had a HDL-c minimum value of 15 mg/dL (media 44 + 15.4) [37]. One potential explanation, which we did not perform in our study, is that it has been reported that Mexican school children aged 6–15 years (*n* = 1253) which carried the C230 allele showed significantly lower HDL-c levels (*p* = 2.9 × 10  (−8)). HDL size was smaller in R230C heterozygotes (*p* < 0.05). These authors suggested that the R230C gene variant plays an important role in HDL-c level regulation in healthy Mexican school-aged children [38]. Furthermore, Acuña-Alonzo et al. found low HDL-c levels were associated with a functional ABCA1 gene variant in Mexican Mestizo population, showing evidence of positive selection [39]. Regarding glucose levels, a study was conducted on 127 children from Sweden, ages 5–7.5 years. Their fasting glucose values ranged from 3.7 mmol/L to 6.1 mmol/L (66.6 mg/dL to 109 mg/dL). So, the range found included low glucose values [40]. Therefore, our statistical analysis and conclusions are not affected by calibration curves if the response is linear.

We also report in this study a significant negative correlation between the irisin levels and physical activity. Underweight children exercised the most; they performed more than twice as much physical activity than the overweight and obese children did. Others have also found that irisin is negatively correlated with physical activities in adults [10, 24] and that no increment in FNDC5 expression occurs in muscle or plasma levels after exercise training [6, 41, 42]. Our results are in accordance, as well, with another study that did not find any evidence of increased irisin secretion after exercise in anorexic adolescents with a severely reduced body weight [43]. A study by Palacios-González et al. also failed to find alterations of the irisin levels with exercise in children [14]. Physical activity stimulates FNDC5 expression, which promotes the browning of white adipose tissue by increasing the expression of uncoupling protein 1 in white adipocytes, increasing thermogenesis and thus energy [4]. Decreased irisin levels in amenorrheic athletes have been described to represent an adaptive response in chronic exercisers for the reduction of energy expenditure and conservation of energy by reducing brown adipogenesis [4]. Lower irisin levels have also been reported in anorexia nervosa [44] and in individuals subjected to weight loss after bariatric surgery [6]. The low irisin levels in the underweight group might also represent an adaptation to reduce energy expenditure and conserve energy, as this group exercised twice as much as the obese group did in the past six months.

The results of this research that was carried out in children contradict those of other studies that have been carried out in adults and that have found positive associations between circulating irisin levels, exercise, and energy expenditure [13, 45, 46]. For example, some studies in adult humans and animals have reported an increase in FNDC5/irisin secretion by adipose tissue after short-term endurance exercise [13]. A possible explanation could be that the type of exercise, either acute or repetitive, as well as the amount or intensity of the activity, could exert diverse effects on irisin secretion, either through the muscle that executes the work or through white adipose tissue that browns as exercise continues, influencing the irisin levels. Most studies included patients subjected to exercise programs, as opposed to ours that used self-reported physical activity.

This study has several limitations. The sample size was small, and the results cannot be extrapolated to other populations or to children, in general. Body muscle mass was calculated with a formula; however, this method is well established, easy to use, helpful in the clinical context [17], and not as stressful for children as a magnetic resonance imaging or a dual energy X-ray absorptiometry scan is. In addition, several articles have questioned the degree of shedding of soluble irisin and the validity of commercially available irisin quantitation ELISA methods; they argue that these methods may be reporting cross-reacting antibodies and a potential null mutation on human FNDC5 [47, 48]. However, this possibility was dismissed in a recent paper using mass spectroscopy to demonstrate that human irisin circulates and is regulated by exercise [5]. In our study, we used the method that has been used by most researchers who evaluate the irisin levels. Another limitation of this study is that we assessed physical activity through a face-to-face questionnaire that we used in a previous publication with a similar population, and this could bias the accuracy of the information obtained. The questionnaire, though, was applied to the children and/or their guardians, face-to-face by a trained person.

This study is the first to examine the association of irisin with adiposity markers, muscle markers, and physical activity in children. This work provides valuable information on irisin in children with a wide range of BMI percentiles, from underweight to obese, by demonstrating the positive association of irisin with adiposity measurements and the lack of one with body muscle mass. It also shows the negative association of irisin with physical activity as well as with metabolic markers. The other strengths of this study were the fact that all children were matched by age and gender for all four groups and that all belonged to the same ethnic group and had the same socioeconomic status; therefore, no racial or socioeconomic differences influenced the results.

## 5. Conclusions

In summary, this study demonstrated that the irisin levels were higher in the obese children than in the underweight and normal weight children. Irisin was positively correlated with adiposity markers, such as the BMI, BMI percentile, WC, and with fat-free mass, but after adjusting for age and gender, only BMI percentile showed a significant positive correlation. Irisin was negatively correlated with body muscle mass. Furthermore, a negative correlation was found between the irisin concentrations and physical activity in children. Irisin showed no association with any of the cardiometabolic markers. The secretion of irisin in children can possibly involve multifactorial regulators under diverse conditions, different from those described for adults, and these conditions can change through the different stages of life and of obesity development. Because conflicting results on the irisin levels have been reported in adults and scarce information in children is available, further research is needed to confirm the association of irisin with different body composition components and physical activity in children. The focus should be on mechanistic studies to clarify the role of irisin in obesity and its influence on physical activity.

## Supplementary Material

Supplemental Table 1. The Pearson correlations of irisin plasma levels with body composition and metabolic parameters are presented in Supplemental Table 2. A significant Pearson correlation was found between irisin levels and BMI percentile (0.387, *p* = 0.01), WC (0.373, *p* = 0.01), and fat-free mass (0.353, *p* = 0.02). Moreover, HDL-c, LDL-c, triglycerides, total cholesterol, glucose, and albumin did not show a significant correlation with irisin levels. Supplemental Table 2. After adjusting for gender, the most significant covariate was BMI percentile. Together with gender, no further covariates were significant. Multiple linear regression analysis indicated that only BMI percentile had a positive correlation with irisin (Supplemental Table 2).

## Figures and Tables

**Table 1:** Demographic, anthropometric, and metabolic parameters.

	Underweight	Normal weight	Overweight	Obesity
Gender	5 (M) 5 (F)	5 (M) 5 (F)	5 (M) 5 (F)	5 (M) 5 (F)
Age (years)	8.5 (7–11)	8.5 (6–12)	8.5 (6–11)	8.0 (6–11)
Height (cm)	126.3 (116–144)	131.4 (113–151)	132 (118–154)	136.4 (123–153)
Body weight (kg)	21.6^(C)(D)^ (17.1–29.5)	29.3^(D)^ (20.5–41.3)	34.6^(B)(D)^ (24.9–49.6)	42.7^(A)(B)(C)^ (33.3–63.4)
BMI (percentile)	3^(C)(D)^ (1–3)	66^(D)^ (35–73)	91^(B)^ (85–93)	98^(A)(B)^ (98-99)
Waist (cm)	50^(A)(C)(D)^ (44–55)	56.8^(B)(C)(D)^ (52–68)	66.8^(A)(B)(D)^ (56–72)	76.5^(A)(B)(C)^ (68–86)
Mild-upper arm circumference (cm)	15.6^(A)(C)(D)^ (14–17.1)	19.1^(B)(C)(D)^ (16.9–22)	21.6^(A)(B)(D)^ (19–26.4)	24.3^(A)(B)(C)^ (21.8–29.5)
Triceps skinfold (mm)	7^(C)(D)^ (4–11)	11.5^(D)^ (7–16)	21^(B)^ (17–31)	33^(A)(B)^ (25–51)
Albumin (g/dL)	5.16 ± 0.09	5.41 ± 0.18	5.06 ± 0.12	5.42 ± 0.12
Glucose (mg/dL)	60.67 ± 3.43	64.15 ± 5.39	53.21 ± 5.78	63.16 ± 4.83
Triglycerides (mg/dL)	54.75 ± 3.57	62.30 ± 7.34	78.81 ± 10.63	86.61 ± 9.08^(A)^
Total cholesterol (mg/dL)	113.15 ± 4.46	115.17 ± 6.13	107.19 ± 5.65	141.35 ± 11.81^(C)^
HDL cholesterol (mg/dL)	16.31 ± 1.3	15.52 ± 1.51	13.66 ± 1.05	14.91 ± 1.32
LDL cholesterol (mg/dL)	85.89 ± 4.57	87.21 ± 6.39	77.77 ± 5.92	109.12 ± 11.14^(C)^

Data presented as median and range; *p* < 0.05 versus ^(A)^normal weight, ^(B)^underweight, ^(C)^overweight, and ^(D)^obesity.

**Table 2:** Association between irisin plasma levels with body composition and metabolic parameters.

	Multiple linear regression (B/*p*)	Pearson correlation (rho/*p*)
BMI (kg/m^2^)	−5.318/0.218	0.307/0.0539
BMI (percentile)	**0.564/0.008**	**0.387/0.0136**
Waist (cm)	−0.409/0.630	**0.373/0.0178**
MUAC (cm)	−4.861/0.163	0.266/0.0970
Fat mass (kg)	3.059/0.273	0.290/0.0691
Fat mass (%)	0.167/0.906	0.276/0.0849
Body muscle mass (kg)	−1.986/0.377	−0.0272/0.868
Fat-free mass (kg)	2.790/0.059	**0.353/0.0254**
Triglycerides (mg/dL)	−0.0623/0.545	−0.0944/0.563
Total cholesterol (mg/dL)	0.202/0.135	0.127/0.435
HDL cholesterol (mg/dL)	0.130/0.855	0.145/0.370

Correlation coefficients are shown for Pearson product and multiple regression analysis as indicated.
