# Supplementary material for: Association of Irisin Plasma Levels with Anthropometric Parameters in Children with Underweight, Normal Weight, Overweight, and Obesity
Source: Biomed Res Int. 2017 May 3;2017:2628968. doi: 10.1155/2017/2628968 (PMC5434279; doi:10.1155/2017/2628968)
Supplement: Supplementary file 1 — Supplemental Table 1. The Pearson correlations of irisin plasma levels with body composition and metabolic parameters are presented in Supplemental Table 2. A significant Pearson correlation was found between irisin levels and BMI percentile (0.387, p = 0.01), WC (0.373, p = 0.01), and fat-free mass (0.353, p = 0.02). Moreover, HDL-c, LDL-c, triglycerides, total cholesterol, glucose, and albumin did not show a significant correlation with irisin levels. Supplemental Table 2. After adjusting for gender, the most significant covariate was BMI percentile. Together with gender, no further covariates were significant. Multiple linear regression analysis indicated that only BMI percentile had a positive correlation with irisin (Supplemental Table 2). [file 2628968.f1.pdf]

SUPPLEMENTAL MATERIAL

Supplemental Table 1. Correlation coefficients between irisin plasma levels and body composition parameters

Supplemental table

	Gender	Age	Height (cm)	Body weight (kg)	BMI (kg/m²)	BMI (percentil)	Waist (cm)	MUAC (cm)	Fat mass (kg)	Fat mass (%)	Body muscle mass (kg)	Fat-free mass (kg)	Glucose (mg/dl)	Albumin (g/dl)	Total cholesterol (mg/dl)	HDL (mg/dl)	LDL (mg/dl)	Triglycerides (mg/dl)	Irisin (ng/mL)
Gender	1	-0.0506	-0.0910	-0.00866	0.0108	-0.0153	-0.0585	0.0693	0.0823	0.143	0.178	-0.0996	-0.0845	0.292	0.0368	-0.483*	0.0520	0.275	-0.418*
Age		1	0.814*	0.436*	0.0824	-0.163	0.246	0.177	0.141	-0.0612	0.481*	0.666*	-0.179	-0.159	0.132	0.208	0.126	-0.0686	0.126
Height (cm)			1	0.795*	0.453*	0.239	0.636*	0.574*	0.540*	0.349*	0.646*	0.917*	-0.216	-0.0931	0.117	0.0626	0.0736	0.162	0.284
Body weight (kg)				1	0.901*	0.662*	0.940*	0.935*	0.934*	0.806*	0.472*	0.929*	-0.169	0.0351	0.267	-0.0806	0.181	0.410*	0.345*
BMI (kg/m²)					1	0.845*	0.934*	0.965*	0.961*	0.942*	0.265	0.714*	-0.0650	0.0977	0.346*	-0.106	0.253	0.464*	0.307
BMI (percentil)						1	0.783*	0.825*	0.736*	0.866*	0.239	0.493*	0.0269	0.0824	0.337*	-0.163	0.247	0.508*	0.387*
Waist (cm)							1	0.947*	0.922*	0.873*	0.379*	0.827*	-0.0820	0.0728	0.386*	-0.129	0.294	0.471*	0.373*
MUAC (cm)								1	0.934*	0.898*	0.447	0.805*	-0.142	0.0578	0.317*	-0.129	0.226	0.451*	0.266
Fat mass (kg)									1	0.935*	0.239	0.735*	-0.126	0.108	0.289	-0.141	0.188	0.507*	0.290
Fat mass (%)										1	0.167	0.560*	-0.0820	0.178	0.291	-0.206	0.193	0.560*	0.276
Body muscle mass (kg)											1	0.648*	-0.192	0.0785	0.231	-0.130	0.177	0.176	-0.027
Fat-free mass (kg)												1	-0.178	-0.0446	0.228	0.00206	0.171	0.250	0.353*
Glucose (mg/dl)													1	0.383*	-0.0247	-0.127	0.00357	0.206	0.0741
Albumin (g/dl)														1	0.192	-0.304	0.200	0.161	-0.0757
Total cholesterol (mg/dl)															1	0.0210	0.968*	0.213	0.201
HDL (mg/dl)																1	0.00310	-0.493*	0.209
LDL (mg/dl)																	1	0.0766	0.208
Triglycerides (mg/dl)																		1	-0.0380
Irisin (ng/mL)																			1

Correlation coefficients are shown, calculated by Pearson product moment or Spearman rank order methods depending on normality of data; $^*p < 0.05$.

Supplemental Table 2. General linear models of variables associated with circulating irisin levels, after controlling for gender and age.

Variables	Main Effect (B/p)
Weight (kg)	1.37/0.104
Height (cm)	0.634/0.155
Plication (mm)	0.296/0.636
Plication Percentile (fraction)	0.094/0.437
Waist (cm)	0.393/0.573
Waist Percentile (fraction)	0.131/0.411
Height-Age Percentile (fraction)	0.144/0.122
**Body Mass Index (kg/m^2)**	**1.36/0.017**
**Body Mass Index Percentile (fraction)**	**0.17/0.005**
Fat percentage (fraction)	0.336/0.653
Body Muscle Mass (kg)	-0.613/0.666
Total body fat (kg)	1.3/0.39
Weekly Activity (days/week)	-1.61/0.14
Daily Activity (hours/day)	-0.445/0.827
Weekly TV (days/week)	-2.16/0.345
Daily TV (hours/day)	1.47/0.4
Mid-upper arm circumference (cm)	-1.51/0.542
Fat free mass (kg)	1.56/0.148
Glucose (mg/dl)	0.066/0.663
Albumin (g/dl)	-0.852/0.879
Total cholesterol (mg/dl)	-0.007/0.946
High Density Lipoprotein (mg/dl)	-0.284/0.668
Triacylglycerides (mg/dl)	-0.078/0.432
